# A Computed Tomographic (CT) and Pathological Study of Equine Cheek Teeth Infundibulae Extracted From Asymptomatic Horses. Part 1: Prevalence, Type and Location of Infundibular Lesions on CT Imaging

**DOI:** 10.3389/fvets.2019.00124

**Published:** 2019-04-25

**Authors:** Apryle Horbal, Sionagh Smith, Padraic M. Dixon

**Affiliations:** The Royal (Dick) School of Veterinary Studies, and The Roslin Institute, The University of Edinburgh, Edinburgh, United Kingdom

**Keywords:** equine dentistry, equine dental imaging, equine infundibular pathology, equine dental pathology, equine dental caries

## Abstract

**Background:** Equine maxillary cheek teeth infundibulae are frequently affected by developmental and acquired disorders, but the computed tomographic (CT) imaging features of normal and abnormal infundibulae remain incompletely understood.

**Objective:** To examine infundibulae with various grades of occlusal caries and control teeth by standard CT in order to assess the prevalence, type and location of subocclusal infundibular lesions present.

**Study design:** *Ex vivo* original study.

**Methods:** One hundred maxillary cheek teeth, including 82 with, and 18 without infundibular occlusal caries, were extracted from horses of different ages and imaged by standard CT; 8 teeth were also imaged by MicroCT. Images were later assessed by Osirix® and the prevalence, characteristics and sites of infundibular lesions were assessed.

**Results:** Teeth with shorter infundibulae (i.e., Triadan 09 position and older teeth) were more likely to have occlusal caries, as were the rostral infundibulae. Subocclusal developmental infundibular lesions, including cemental hypoplasia and caries, were present in 72% of infundibulae without occlusal caries. CT imaging confirmed two main patterns of developmental cemental hypoplasia, i.e., *apical cemental hypoplasia* usually involving the full width of the apical aspect of the infundibulum and *central linear hypoplasia* involving the central aspect of the infundibulum over most of its length, and combinations of these types. These developmental lesions could later be affected by (acquired) infundibular caries once occlusally exposed due to normal wear. Some “normal-sized” (i.e., circa 1 mm diameter) occlusal *central vascular channels* expanded subocclusally to the dimensions of central linear defects.

**Main Limitations:** No clinical histories or accurate ages were available for these teeth.

**Conclusions:** Hypoplastic cemental lesions, including at central linear, and apical sites, are common even in clinically normal maxillary cheek teeth infundibulae and caries can occur when these lesions contact the occlusal surface. Central linear defects are not always clearly distinguishable from “normal” central vascular channels.

## Introduction

Equine maxillary cheek teeth each contain two infundibulae that provide additional occlusal enamel ridges to help crush the fibrous, cellulose-containing equine diet. Anatomical (1, 2) and computed tomographic (CT) studies (3–6) have shown that up to 90% of equine cheek teeth infundibulae, in particular the rostral (mesial) infundibulae of the Triadan 09 position, are incompletely filled with normal cementum, that ideally should be present. These infundibular cemental defects, mainly developmental in origin, include the very common presence of a small central cemental defect, the site of a previous blood vessel variously termed the “vascular channel,” “central vascular channel,” or “central linear defect” for much of the length of the infundibulum. More clinically important are larger areas with less-dense, usually discolored hypoplastic cementum or even total absence (aplasia) of cementum, centrally and/or toward the apical aspect of infundibulae (1, 2, 7). Caries of infundibular cementum is an acquired, bacterial-acid mediated disorder, that appears to preferentially affect infundibulae with developmental defects that trap food (8–10). Infundibular cemental caries can later extend to the adjacent infundibular enamel, then even to dentine and pulp (2, 7, 9–14).

Maxillary cheek teeth, especially the Triadan 09 position, are prone to develop midline sagittal fractures that were initially described as a variant of “idiopathic cheek teeth fractures” (15). However, after their aetiopathogenesis became apparent by recognizing that they invariably had coalescing infundibular caries, they were later termed *caries-related infundibular fractures* (12). Additionally, if infundibular caries penetrates through infundibular enamel and dentine to an adjacent pulp, it can cause apical infection without development of a dental fracture (7, 11, 14).

Suske et al. have recently described the gross and histological findings in maxillary cheek tooth infundibulae with various developmental and acquired cemental defects that predisposed to serious clinical disorders (7). A recent epidemiological study of infundibular caries found no major dietary predispositions to infundibular caries, but a strong association with the presence of concurrent peripheral caries (16). Despite these studies, our knowledge of equine cheek teeth infundibulae in health and disease remains incomplete. The aim of this study was to examine the CT characteristics of cheek teeth infundibulae with and without occlusal infundibular caries in horses of different ages.

## Materials and Methods

Cadaver heads without clinical histories and obtained from an abattoir were disarticulated at the temporomandibular joint. The oral cavity was lavaged with water to allow visual examination of the maxillary cheek teeth occlusal surfaces and to also allow approximate aging of the heads by incisor examination, supplemented by later examination of extracted cheek teeth. The age of the horse rather than the *dental age* of extracted teeth was recorded. To reduce the potential inaccuracy of aging by dental examination, age was recorded in 5-year ranges. i.e., <5 years old (y.o.); 5–10; 10–15; 15–20; and >20 y.o.

**Figure 1:**
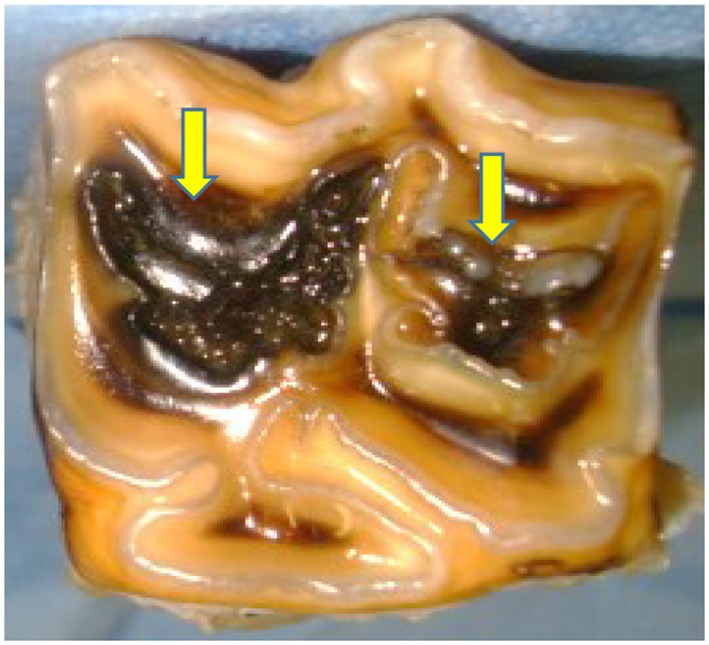
Grade 3 caries of the rostral (mesial) infundibulum (on left side) involving infundibular cementum (black or missing), enamel (black), and bucally located (discolored) dentine (arrow). Limited enamel caries of the caudal (distal) infundibulum (right side), with a slight area of discolored dentine (arrow), therefore a Grade 3 caries. (x2).

Currently, there are no universally accepted guidelines to assess maxillary cheek teeth infundibular cemental defects. In this study, guidelines adapted from those of Suske et al. (3, 7) were used to distinguish between “normal” cemental central vascular channels, i.e. single, centrally located, occlusal cemental defects *circa* 1 mm in diameter without surrounding discolored/brittle cementum, from occlusal infundibular caries (i.e. central cemental defects of >1 mm diameter with surrounding discolored/brittle cementum). Occlusal infundibular caries was graded using the modified Honma criteria (16, 17), i.e., Grade 1 = cemental caries; Grade 2 = cemental and enamel caries; Grade 3 = cemental, enamel and dentinal caries; Grade 4 = loss of dental structural integrity i.e., coalesced carious infundibulae or sagittal fracture. An example of this grading system in two carious infundibulae is shown in Figure 1.

### Selection of Teeth

Thirty heads without evidence of sinusitis and containing cheek teeth without major dental disorders such as dysplasia, fractures or pulp exposure were selected from horses of the five different age groups. Within these age groups, maxillary cheek teeth with occlusal infundibular caries lesions were selected without regard for Triadan position including from horses <5 y.o. (*n* = 14 teeth); 5–10 y.o. (*n* = 27); 10–15 y.o. (*n* = 19); 15–20 y.o. (*n* = 23); >20 y.o. An attempt was then made to select control teeth from matching Triadan positions to the teeth with occlusal infundibular caries. The limited availability of horses <5 y.o. necessitated 12 of 14 teeth in this age group being taken from one 3.5 y.o. horse. Only two of the 30 skulls (6.7%) were free of occlusal infundibular lesions in all teeth and consequently, 16 of the 18 control cheek teeth were obtained from cadavers which had other cheek teeth affected by occlusal infundibular caries. Older teeth with worn out infundibulae (“senile excavation”) were excluded from the study, that additionally limited the availability of aged (>20 y.o.) teeth. A mean of 3 teeth (range 1–12 teeth) per head were extracted.

The 100 selected cheek teeth included 18 teeth (36 infundibulae) without occlusal caries of either infundibulum (control teeth), and 82 teeth with occlusal infundibular caries, affecting the rostral (mesial) infundibulae only (*n* = 18); the caudal (distal) infundibulae only (*n* = 10) or both infundibulae (*n* = 54); i.e., a total of 136/164 infundibulae in these 82 teeth were affected with occlusal caries, and 28 infundibulae had no occlusal caries All teeth were extracted intact where possible, using a steel osteotome and mallet and were placed in 10% buffered formalin solution until further examined.

### Standard Computed Tomographic Imaging

Standard CT imaging was performed on all extracted teeth with a multi-slice scanner (Siemens® SOMATOM Volume Zoom, Siemens, Munich, Germany) using a 512 9 512 matrix, 120 kV, 300 mA, at a slice thickness of 0.5 mm with 0.5 mm overlap. Bone window CT data were transferred as DICOM images to imaging software (OsiriX^©^). Measurements of total crown (enamel containing areas) length (CL) and infundibular depth (ID) were compiled from the CT scans.

### Micro Computed Tomographic (MicroCT) Imaging

Micro computed tomographic (MicroCT) scanning (XTreme CT, Scanco Medical AG, Bruttisellen, Switzerland) at an isotropic spatial resolution of 82 micrometers was additionally performed on 8 of the above cheek teeth, including Triadan 08 (*n* = 1), 09 (*n* = 6), 10 (*n* = 1) from age groups <5 y.o. (*n* = 2); 5–10 y.o. (*n* = 2); 10–15 y.o. (*n* = 1); 15–20 y.o. (*n* = 2); >20 y.o. (*n* = 1). Occlusal caries was present in 13 of the 16 infundibulae of these teeth, including grade 1 (*n* = 10), grade 2 (*n* = 1), and grade 3 (*n* = 2) caries. Acquired images were later examined using OsiriX^©^ software.

### Characteristics of Subocclusal Infundibular Lesions Identified by Standard Computed Tomography Imaging

**Figure 2:**
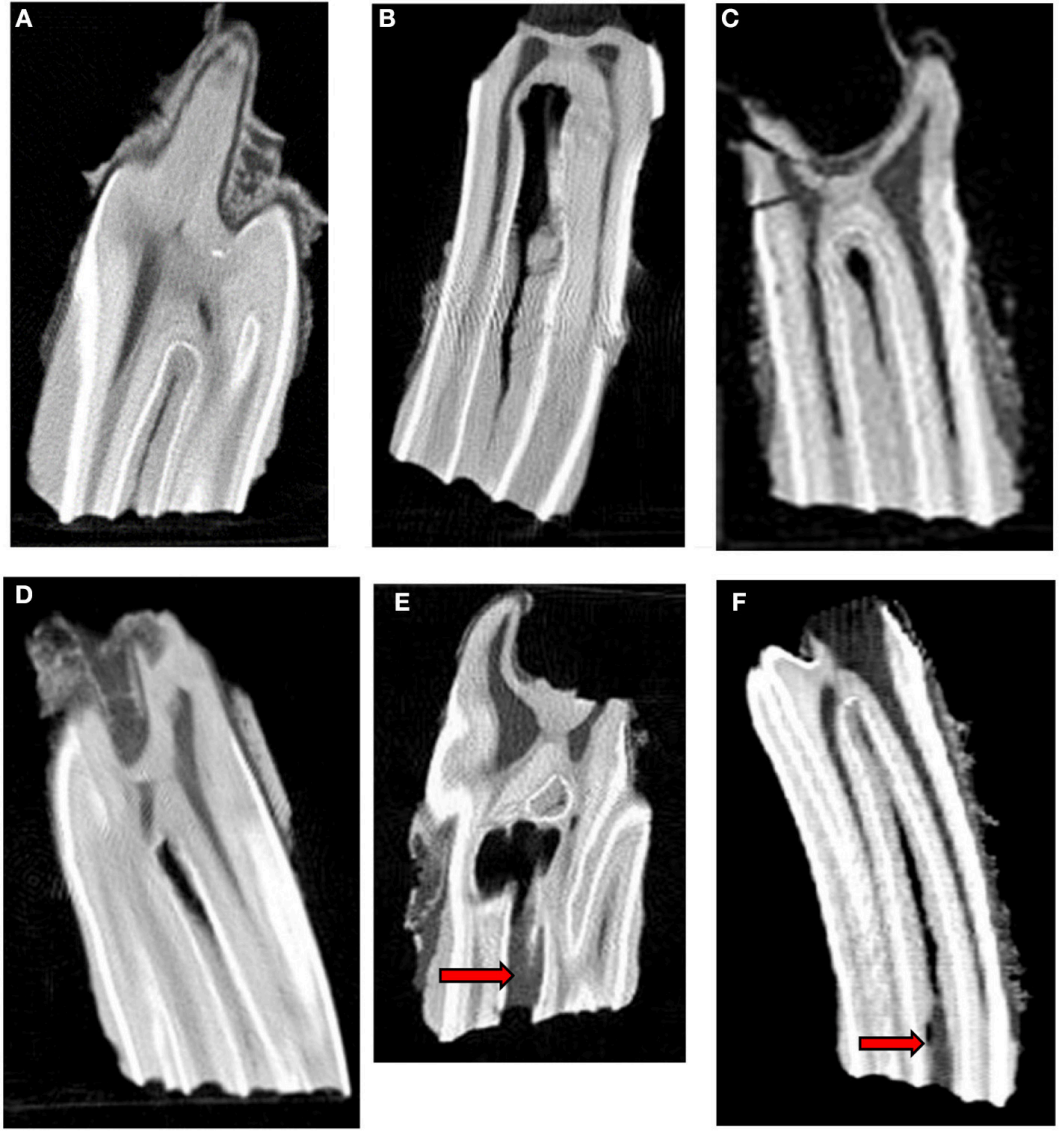
Longitudinal standard CT images of 6 infundibular lesions. **(A)** Longitudinal CT image of an older tooth showing a fine central linear cemental defect. This defect appears slightly wider than the “normal” central vascular channel. **(B)** Longitudinal CT image of a young tooth shows a central linear cemental defect (2–5 mm wide) continuous with marked apical cemental hypoplasia. There is no occlusal cemental defect and consequently no subocclusal caries at this stage. **(C)** Longitudinal CT image of an older tooth showing localized apical cemental hypoplasia extending occlusally as a linear type central defect to about 50% of infundibular depth **(D)** Longitudinal CT image of an older tooth shows localized apical cemental hypoplasia/aplasia, with a thin oblique layer of hypoplastic cement between it and the normal appearing cementum. **(E)** Longitudinal CT image of an older tooth. Occlusal caries is continuous with extensive subocclusal infundibular caries (arrow) and to an apical diverticulum with marked/total cemental hypoplasia that would later become filled with food. **(F)** Longitudinal CT image of a young tooth showing a wide hypointense, central linear cemental defect connected to an occlusal caries lesion (arrows) of soft tissue density.

A review of the literature (1–5, 7, 12) and pilot studies (Horbal and Dixon 2014, unpublished observations) showed that subocclusal infundibular lesions could most readily be classified on CT imaging into 4 different categories. The following guidelines were used to characterize infundibular CT findings in this study (Figures 2A–F).

1. Central vascular channel is a fine (circa 1 mm diameter) linear, subocclusal central cemental defect that is the site of the former cemental vasculature. This structure (if present) was readily detected on all transverse images (although not visible on most longitudinal planes). It is so commonly found clinically, as well as on imaging that it is widely considered as normal variation (Figure 2A).
2. Central linear cemental defects (Figures 2B,F) comprised a much wider (typically 2–5 mm in diameter) linear central channel, as opposed to a central vascular channel remnants (circa 1 mm diameter). Central linear defects can be concurrently present with deeper apical cemental hypoplastic defects (Figures 2B,F).
3. Apical cemental hypoplasia was identified when all or most of the width of the infundibulum was incompletely filled with normal cementum toward the apical aspect of infundibulae (Figure 2B). However, in some cases, these lesions can extend occlusally over most of the length of infundibulum and they can be concurrently present with central linear defects (Figures 2B,E).
4. Subocclusal infundibular caries was an extension of occlusal infundibular caries deeper into the infundibulum (Figures 2E,F). Occlusal caries was characterized on CT imaging as central cemental defects that were wider than the “normal” vascular channel that always directly communicated with the occlusal surface. In this study, subocclusal caries was subdivided into shallow (< 10 mm deep) carious lesions, with all or most of the carious lesion recognizable from the occlusal surface, and deeper (>10 mm deep) carious lesions that were a direct, deeper extension of occlusal caries.

### Statistical Techniques

Most continuous data obtained were non-normally distributed as determined by use of probability plot analysis (Minitab 17.1.0®, Minitab Inc, State College, Pennsylvania, USA) and were analyzed using the Wilcoxon Signed Rank test, including for difference in occlusal infundibular caries grades and infundibular depths between the rostral and caudal infundibulae.

For qualitative data analyses, Chi-square test were used to analyse nominal data including relationships between the presence of occlusal infundibular caries and Triadan position, and the frequency of subocclusal lesions in different age groups. The Kruskal-Wallis test was used to analyse ordinal data including infundibular depth: crown length ratios between age groups; Triadan positions and grade of occlusal infundibular caries. Most statistical calculations were made using Minitab 17.1.0® software and Stata 14^©^ (StataCorp LP, College Station, Texas, USA).

## Results

### Occlusal Infundibular Caries Grade

As noted, 28 infundibulae in the 82 teeth affected with occlusal caries (affecting one or both infundibulae), in addition to all 36 infundibulae in the 18 control teeth had no occlusal caries (total number of infundibulae without occlusal caries = 64). The 136 infundibulae with occlusal infundibular caries included 84 (61.8%) with Grade 1; 38 (27.9%) with Grade 2; and 14 (10.3%) with Grade 3 caries.

In the 82 teeth with occlusal infundibular caries of one or both infundibulae, the rostral infundibulae had grade 0 (*n* = 10; 12.2%); grade 1 (*n* = 40; 48.8%), grade 2 (*n* = 24; 29.3%), grade 3 (*n* = 8; 9.8%) occlusal infundibular caries, whilst their 82 caudal infundibulae had grade 0 (*n* = 18; 22.0%); grade 1 (*n* = 44; 53.7%); grade 2 (*n* = 14; 17.1%); grade 3 (*n* = 6; 7.3%) infundibular caries.

Overall, the rostral infundibulae were more frequently affected with occlusal caries (87.8%) than the caudal infundibulae (78% affected). The same *grade of occlusal caries* was present in both infundibulae in 52% of teeth, the rostral infundibulae had higher grades of caries in 34% and the caudal infundibulae had higher grades in 14% of teeth. When the rostral and caudal infundibulae were paired by tooth and the differences in their occlusal caries grade tested, the rostral infundibulae were significantly more frequently affected by higher grades of caries than the caudal infundibulae (*p* = 0.02).

### Relationship Between Occlusal Infundibular Caries and Triadan Position

**Figure 3:**
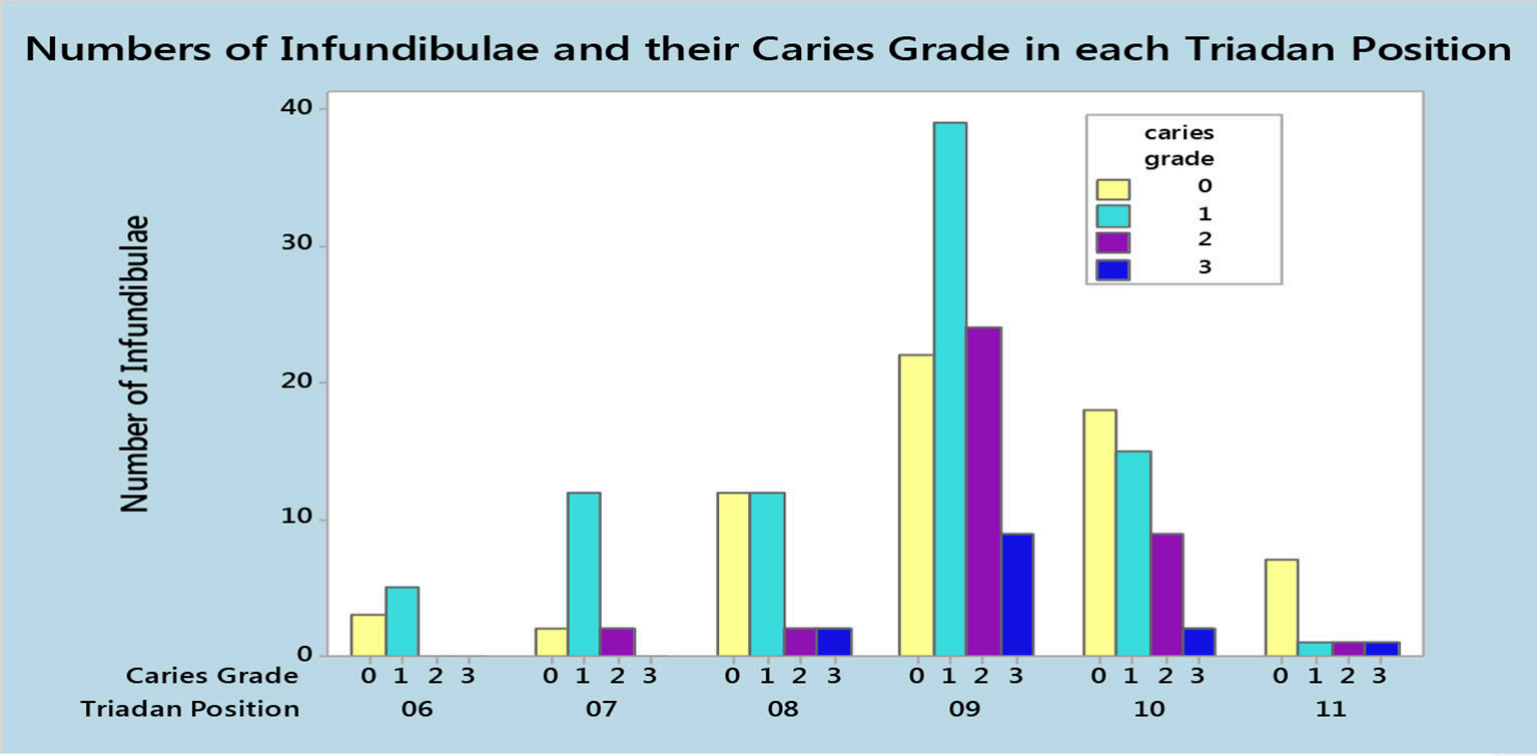
Number of infundibulae and the grades of occlusal infundibular caries in each Triadan position in 100 maxillary cheek teeth, including 82 teeth with caries in one or both infundibulae and 18 teeth without occlusal infundibular caries.

The 82 cheek teeth with occlusal infundibular caries included: Triadan 09s (*n* = 41; 50%); 10s (*n* = 17; 21%); 08s (*n* = 10; 12%); 07s (*n* = 8; 10%); 06s (*n* = 3; 4%), and 11s (*n* = 3; 4%). The 18 control teeth included; Triadan 09s (*n* = 6; 33%); 10s (*n* = 5; 28%); 08s (*n* = 4; 22%); 11s (*n* = 2; 11%), and 06s (*n* = 1; 6%) (Figure 3). The Triadan 09 position comprised 50% of affected teeth, resulting in a statistically significant correlation between the presence of occlusal infundibular caries and the Triadan position of affected teeth (*p* = 0.012).

### Standard Computed Tomographic Findings

#### Infundibular Depth Measurements

The median crown length (CL) of all 100 teeth was 46.4 mm (mean 50.4 mm, range 17.2–82.1 mm), whilst median infundibular depth (ID) (*n* = 200) was 25.6 mm (mean 33.0 mm; range 0–74.4 mm). The median ID:CL ratio of all 200 infundibulae was 0.59 ranges (range 0–0.97); of the 100 rostral infundibulae was 0.56 and of the 100 caudal infundibulae was 0.61, showing that over all age groups, the caudal infundibulae were slightly longer than the rostral infundibulae (*P* < 0.001). Infundibulae were a median of 88% of crown length in horses <5 y.o; 81% in 5–10 y.o.; 53% in 10–15 y.o.; 46% in 15–20 y.o.; and 39% in >20 y.o. This age-related reduction in ID:CL ratio was significant (*P* < 0.001).

#### Relationship Between ID:CL Ratio and Triadan Position

The ID:CL ratio also differed significantly (*p* = 0.033) between Triadan positions (Triadan 06 = 0.66; Triadan 07 = 0.82; Triadan 08 = 0.61; Triadan 09 = 0.57; Triadan 10 = 0.58; Triadan 11 = 0.61).

#### Relationship Between ID:CL Ratio and Grade of Occlusal Infundibular Caries

There was also a statistically significant difference between the median ID:CL ratio of cheek teeth with different grades of occlusal infundibular caries (*p* < 0.001).The median ID:CL ratio decreased from 0.74 in grade 0 caries (*n* = 64); 0.63 in grade 1 caries (*N* = 84); 0.57 in grade 2 caries (*n* = 38) to 0.44 in grade 3 caries (*n* = 14).

#### Prevalence of Subocclusal Infundibular Lesions

Infundibular lesions detected on CT imaging included more occlusally located lesions (that were also visible grossly as occlusal caries) and deeper subocclusal lesions only visible on imaging. Of 200 infundibulae examined by standard CT, including 64 infundibulae without occlusal infundibular caries, 182 (91%) had some type of *subocclusal* cemental lesion. These subocclusal lesions were present in both infundibulae in 86% (86/100) of teeth and in just one infundibulum in 10% (10/100) of teeth. Only 4 (4%) teeth had no subocclusal infundibular lesions.

Types of Subocclusal infundibular lesions included:

7.5% (*n* = 15) with shallow caries localized to < 10 mm below the occlusal surface
27% (*n* = 54) with deeper (>10 mm) subocclusal caries along with apparent concurrent central linear defect and/or cemental hypoplasia in all cases (Figures 2E,F)
32% (*n* = 64) with central linear cemental defects only
21% (*n* = 42) with central linear defects and apical cemental hypoplasia
3.5% (*n* = 7) with apical cemental hypoplasia only
9% (*n* = 18) with no identified lesion—other than a fine central “vascular channel” in the occlusal aspect of infundibular cementum (all 18 infundibulae were from the 18 control teeth).

The numbers and types of subocclusal infundibular lesions in relation to the presence or absence of occlusal infundibular caries and in the different age groups are presented in Tables 1, 2, respectively. Occlusal caries was not recognized on CT imaging in 67 infundibulae with clinically recognized shallow occlusal caries lesions, but each of these infundibulae had some form of cemental hypoplasia.

**Table 1:** Number and types of subocclusal lesions present in 200 infundibulae in relation to the presence or absence of occlusal infundibular caries.

**Subocclusal lesion**	**Occlusal caries Present**	**No occlusal caries**	**Total**
No lesion	0	18	18
Caries < 10 mm deep	15	0	15
Caries >10 mm deep	54	0	54
Central linear defect (CLD)	41	23	64
Apical cemental hypoplasia (ACH)	2	5	7
CLD + ACH	24	18	42
Total	136	64	200

**Table 2:** Number and types of subocclusal lesions in 200 infundibulae of 5 different age groups.

**Age group (in years)**	**<5**	**5–10**	**10–15**	**15–20**	**>20**	**Total**
No lesion	4	2	2	6	4	18
Caries < 10 mm deep	0	2	2	7	4	15
Caries >10 mm deep	0	10	15	16	13	54
Central linear defect (CLD)	15	24	10	7	8	64
Apical cemental hypoplasia (ACH)	0	0	2	2	3	7
CLD + ACH	9	16	7	8	2	42
Total	28	54	39	46	34	200

As noted, CT changes were recognized in 182 of 200 infundibulae, including 136 infundibulae with, and 46 infundibulae without occlusal caries. The latter 46 infundibulae (including 18 of 36 infundibulae in the 18 control teeth) all had developmental cemental defects, i.e., central linear cemental defects and/or apical cemental hypoplasia that did not have (at the stage of eruption when the teeth were extracted) a sufficient direct connection with the occlusal surface to allow food and oral bacterial contamination of the subocclusal defect with the likely subsequent development of recognizable occlusal infundibular caries.

All the deep (>10 mm) carious lesions (Figures 2E,F) appeared to have some form of concurrent cemental hypoplasia, but some cemental loss would also be caused by caries.

The rostral infundibulae had lesions in 94% of teeth, while the caudal infundibulae had lesions in 88% of teeth. All infundibulae affected by occlusal caries all had subocclusal lesions, whilst 75% of infundibulae without occlusal caries had subocclusal lesions (all cemental hypoplasia).

Due to the high prevalence of subocclusal infundibular lesions in these selected teeth, with an 86–100% prevalence in the different Triadan positions and 85–96% prevalence in the different age groups, there was no statistically significant difference between the prevalence of these lesions between the different Triadan positions or age groups (*p* = 0.77).

#### MicroCT Findings

**Figure 4:**
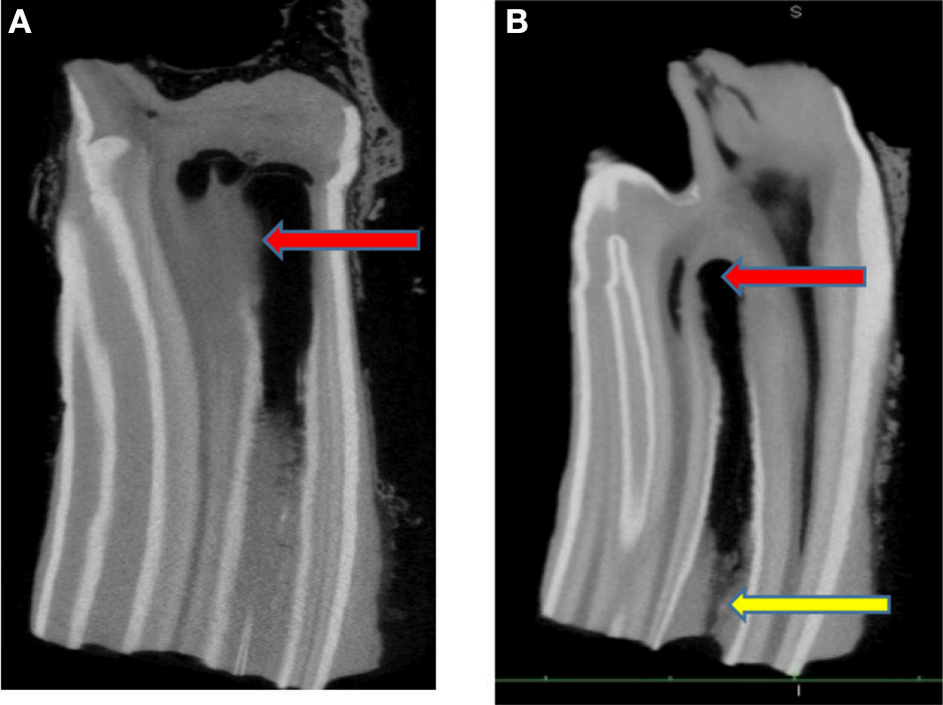
**(A)** Longitudinal microCT image of a maxillary cheek tooth with Grade 2 caries of its rostral infundibulum that has an apical diverticulum, as evidenced by loss of definition of the apical infundibular enamel, with an irregular and hypointense outline of its site visible (arrow). Some of surrounding dentine seems hypointense indicating it also may have changes, (possible Grade 3 caries). The subocclusal defect that connected with and allowed oral bacteria to access this site is not shown in this imaging plane. x1. **(B)** Longitudinal microCT image of a maxillary cheek tooth with grade 2 caries (cement and enamel involvement) of its rostral infundibulum (following central linear defect and/or apical cemental hypoplasia). The apical dentine appears normal. The occlusal defect allowing extension of occlusal caries to this apical site is visible (yellow arrow). x 1.

**Figure 5:**
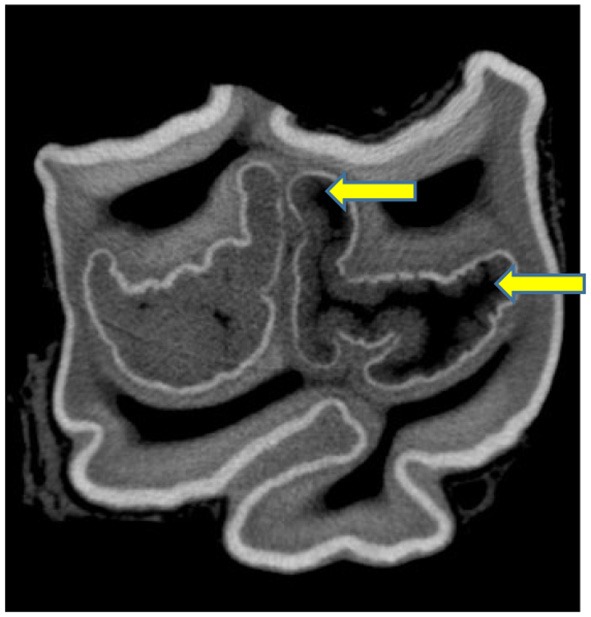
Transverse microCT image of a maxillary cheek tooth. Two small (likely clinically insignificant) cemental defects are present centrally in the caudal infundibulum (on left). Extensive apical cemental hypoplasia (mainly centrally) is present in the rostral infundibulum (on right) but also in its buccal infoldings (arrows). (x 2 when width in bucco palatal plane = 4.5 cm).

**Figure 6:**
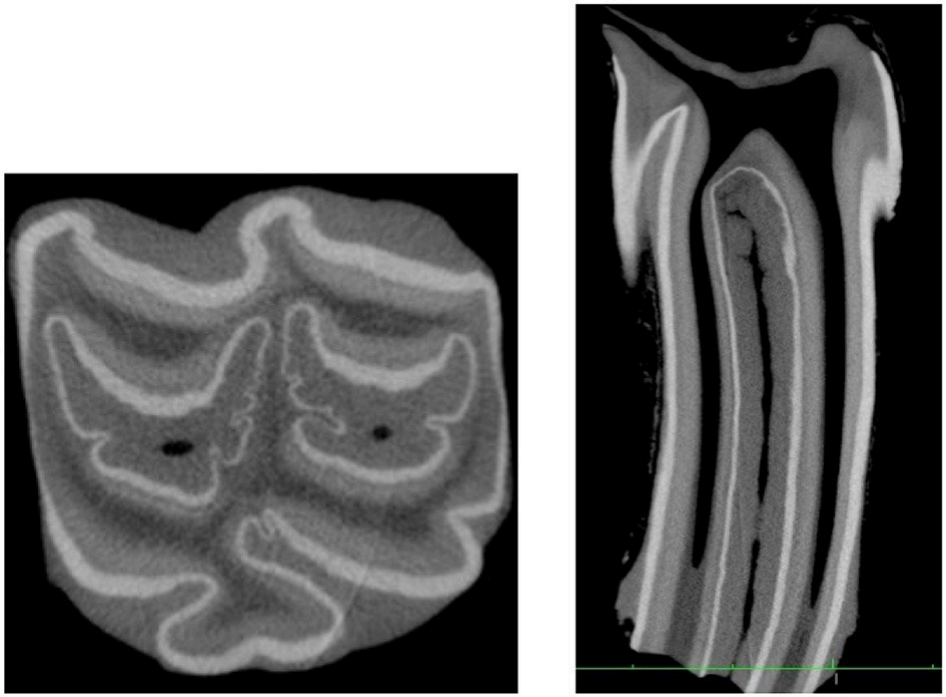
Transverse (x2 magnification, on left) and longitudinal (no magnification, on right) microCT images of the same cheek tooth with small (circa 1 mm wide in the palato-buccal plane) occlusal central linear cemental defects of both infundibulae on the transverse image, considered as innocuous “central vascular channels.” However, at other subocclusal sites on the longitudinal image, one of these central linear defects is up to 3 mm wide (X2 magnification for transverse image when width in bucco-palatal plane = 4.4 cm; X1 magnification for longitudinal image).

**Figure 7:**
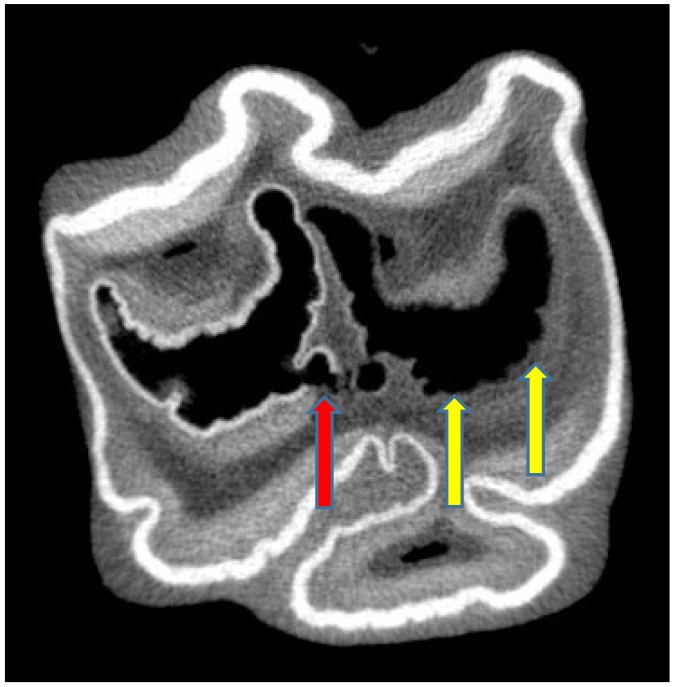
Subocclusal, transverse microCT image of a tooth affected with Grade 2 infundibular caries and also with a small area of possible grade 3 caries (adjacent dentine also affected) (red arrow) of caudal infundibulum (on left). More extensive Grade 3 caries is present in the rostral infundibulum (on right) (yellow arrows). x 2.

**Figure 8:**
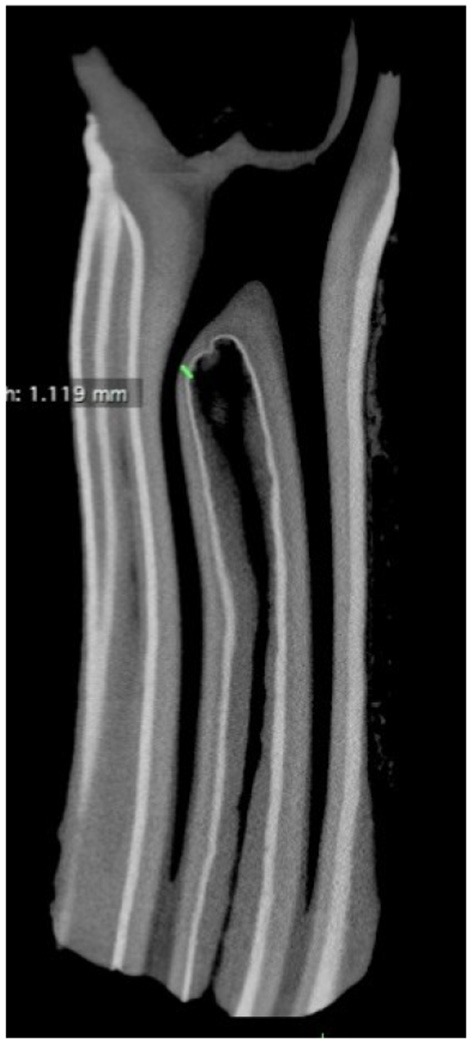
Transverse microCT image of infundibulum with combined central linear defect and apical cemental hypoplasia. Note the close proximity of the infundibulum and pulp horn at a site of thin infundibular enamel and adjacent thin dentine (green line). x 1.

The median length of: the 8 crowns imaged by microCT was 56.3 mm (range 54.0–79.1 mm); the rostral infundibulae was 38.4 mm (range 19.5–65.4 mm) and caudal infundibulae was 36.2 mm (range 21.7–69.2 mm). MicroCT scan reconstructions in both the longitudinal and transverse planes of these teeth confirmed the presence of the infundibular lesions imaged on conventional CT in all teeth. As expected, the thinner image slices of MicroCT showed much greater anatomical detail (Figures 4–8). Subocclusal infundibular lesions were present in 15/16 infundibulae of these 8 teeth including: apical cemental hypoplasia combined with central linear defects (*n* = 7); central linear defects (*n* = 4); deep infundibular caries with apical cemental hypoplasia and/or central linear defects (*n* = 3); apical cemental hypoplasia (*n* = 1) (Figures 4–8). Only one infundibulum had complete cemental filling. Infundibulae with advanced caries additionally had enamel changes, including loss of density of enamel, and/or a scalloped or even total loss of its outline that indicated grade 2 infundibular caries, but early dentinal caries was less obvious (Figures 4A,B, 7).

## Discussion

### Gross Infundibular Occlusal Findings

Because 28 of the 30 heads used in this study were selected on the basis of having occlusal caries of one or both infundibulae, the overall prevalence of occlusal caries of 93.3% obtained in this study is a gross overestimate of its prevalence in the current population. Not surprisingly, it is higher than values obtained from surveys of the general equine population such as the 45.5% prevalence recorded by Borkent et al. (16).

Additionally, an attempt was made in this study to obtain equal numbers of teeth from five different age groups, which also precludes any age-related statistical analysis of the findings. However, it is still interesting to note that teeth from the youngest (< 5 years old) age group were unaffected by Grade 2 and 3 *occlusal* caries. This is likely because the developmental infundibular cemental defects present in 24/28 (86%) infundibulae these young teeth had not yet worn to a level that would allow significant communication of the cemental defects with the oral cavity. Conversely, some teeth from the oldest age groups were completely unaffected by occlusal caries, showing that some teeth are resistant to infundibular caries formation, if their infundibulae became completely filled with normal-appearing cementum during dental development.

Although the 82 affected teeth were selected for the presence of caries without regard to their Triadan position, the 09 Triadan position (comprised 41/82 i.e., 50% of affected teeth) was 5-fold overrepresented as compared to the other 5 Triadan positions. The 09 position has previously been shown to be the Triadan position most commonly affected by infundibular caries (2–4, 7, 12, 13). In contrast, the Triadan 06 and 11 positions were least affected by infundibular caries in this study, as also previously found (2, 7).

Occlusal caries was mainly (62%) confined to infundibular cementum (i.e., Grade 1), with 28% Grade 2, and 10% Grade 3 caries affected infundibulae. The grade of occlusal caries also differed significantly between Triadan positions, with the high proportion of affected Triadan 09s likely influencing this finding. In the current study, the 09 position had a higher proportion of Grade 2 and 3 caries in comparison to the other Triadan positions, while the 06, 07, and 11 teeth were rarely affected by Grade 2 or 3 caries (Figure 3).

### Infundibular Depth:Crown Length (ID:CL)

The ID:CL ratio decreased in older horses as the crown wore and the infundibulae became proportionally shorter in relation to crown length; from a median of 0.88 in horses < 5 y.o. to 0.41 in horses >20 years old. Likewise, Fitzgibbon et al. found the ID:CL ratio to decrease from 0.99 in a 4 y.o. horse to just 0.07 in a 16y.o horse (2). In the current study, the ID:CL ratio was, as expected, lowest (0.57) in the Triadan 09 position (the oldest permanent tooth). Additionally, the ID:CL ratio decreased as the grade of occlusal caries increased, from 0.74 in unaffected teeth to 0.44 in infundibulae with Grade 3 caries, that usually affected older teeth. The high proportion of 09 infundibulae (shorter crowns) with Grade 3 caries also contributed to this result.

### Prevalence of Subocclusal Lesions on Standard CT Imaging

Clinically, the occlusal surfaces of both infundibulae were normal in the 18 control teeth in this study (i.e., a non-carious, central cemental defect circa 1 mm diameter). However, standard CT imaging showed that 50% (18/36) of infundibulae in these control teeth had some type of developmental subocclusal cemental defect, as did all 28 infundibulae without occlusal caries in the 28 teeth where only one infundibulum was affected with occlusal caries. These defects comprised a central linear defect and/or apical cementum hypoplasia, without the presence of caries. With normal occlusal wear and dental eruption, these deeper cemental lesions would eventually become directly exposed on the occlusal surface and along with the presence of appropriate cariogenic oral microbiota and diet, these infundibulae would then have developed occlusal and subocclusal caries (1, 7). This finding further highlights the limitations of using clinical assessment at a single time point in younger horses to assess for the presence of such a progressive, age-related disease (16).

Many authors (2–5) have shown that the rostral infundibulae of maxillary cheek teeth are more likely to be affected by developmental cemental defects and caries than the caudal infundibulae. The current study, albeit on selected teeth, also found the rostral infundibulae more commonly affected (72% prevalence) with cemental lesions than the caudal infundibulae (62% prevalence), although this difference was not statistically significant. However, the rostral infundibulae did have a significantly higher grade of caries than the caudal infundibulae. This increased prevalence and severity of rostral infundibular lesions is likely related to the earlier loss of the lateral accessory blood supply to the rostral as compared to the caudal infundibulum (3) limiting post-eruption cementogenesis in the former.

### Standard Computed Tomographic Classification of Infundibular Lesions

Vera et al. described infundibular hypoattenuation, i.e., reduced radiodensity, in 85% of the Triadan 08, 09, and 10 infundibulae they imaged by CT, mainly affecting the apical aspect of infundibulae (4). Windley et al. (5) categorized infundibular cemental defects detected by CT imaging into cemental hypoplasia—both at the amelocemental junction, as histologically described by Kilic et al. (1); and also centrally around the site of the former vascular channel, and (acquired) infundibular caries. All major cemental hypoplasia lesions in the current study involved the central aspect of infundibulae, but in infundibulae with apical cemental hypoplasia, the cemental hypoplasia usually extended fully to the peripheral walls.

Small central linear cemental defects (central vascular channels) arbitrarily stated to be up to 1 mm diameter on the occlusal surface, are currently considered non-significant, However these small cemental channels were found to become wider subocclusally in some teeth. For example, the central cemental defect shown in Figure 6 was circa 1 mm diameter occlusally but expanded to 3 mm diameter subocclusally, where it would then be classified as a central linear defect. There appears to be no absolute distinction between “normal sized” central vascular channels and central linear developmental cemental defects, and further research in this area is needed.

Apical cemental hypoplasia was most commonly found in infundibulae concurrently affected by other lesions including central linear defects (21%) and/or deeper (>10 mm) depth infundibular caries, where it and/or central linear defects appeared to be present in all cases (27% of infundibulae). However, it is possible that extensive carious destruction of cementum was present in some infundibulae with advanced caries. Apical cemental hypoplasia was rarely (3.5% prevalence) detected deep to otherwise normal cementum. Apical cemental hypoplasia, by definition, involved the apical aspect of infundibulae, usually the apical third, but when combined with a concurrent central linear defects and/or caries, this type of defect could have a direct connection with the occlusal surface. Therefore, the classification of the two main types of cemental hypoplasia, i.e., central linear lesions and apical cemental hypoplasia is indistinct in some teeth, with many variations and combinations of these cemental lesions. Their classification is further complicated when they became occlusally exposed and deeper infundibular caries with further cemental destruction 24 develops.

There was a steady, age-related decline in the proportion of infundibulae affected by any type of infundibular cemental hypoplasia, with the highest prevalence in the < 5 year age group (86%) and the lowest in the >20 year group (38%). As noted above, this age-related decrease in prevalence is likely due to areas of apical cemental hypoplasia later becoming exposed on the occlusal surface due to dental wear and then developing caries. Conversely, subocclusal caries increased from 0% prevalence in the < 5 y.o. group to 50% in the 15–20 and >20 y.o. groups.

Subocclusal cemental (Grade 1) caries was readily identified on CT when hypoattenuated infundibular cementum had an obvious connection to the occlusal surface (Figures 2E,F, 4B). However shallow occlusal caries can be difficult to identify because the softer infundibular cementum always wears faster than the adjacent harder enamel, always causing a depression of the occlusal cementum that is similar to very localized occlusal caries. Consequently, superficial Grade 1 occlusal caries was not detected on CT in 67 infundibulae or was found to be very superficial (*n* = 15). Deeper (>10 mm) Grade 1 (cemental) caries appeared as hypointense areas in comparison to normal cementum, due to the presence of degraded carious cementum and/or developmentally porous cementum along with impacted food material. However, areas of cemental caries were usually hyperintense in comparison to areas with marked cemental hypoplasia or aplasia (the latter sometimes partially filled with gas). Caries of infundibular enamel (adjacent to carious cementum) was recognized on CT by its loss of density initially and, later, even loss of its outline (Figures 4B, 7), with some teeth also having changes in the adjacent dentine (Grade 3 caries) (Figure 7) that may have been dental sclerosis rather than carious destruction.

Cemental hypoplasia, as described above, should not be confused with incomplete development of all of the infundibular apex including its enamel, which allows direct communication between the oral cavity and the apical aspect of the periodontium, leading to apical periodontal infection, as described in a small number of cases (11, 18, 19).

Focal defects in infundibular enamel that could allow cemental caries to more readily extend to adjacent dentine and pulp horns have been described by some authors (7, 17), but these were not observed in this study, although some areas of very thin infundibular enamel were identified (Figure 8).

## Conclusions

This study confirmed the predisposition of the 09 Triadan position to develop occlusal infundibular caries and for the rostral infundibulae to be more commonly and severely affected with occlusal infundibular caries than the caudal infundibulae. Relatively shorter infundibulae (older teeth and Triadan 09s) were more likely to have occlusal caries. There was a high prevalence of subocclusal infundibular lesions, especially cemental hypoplasia in maxillary cheek teeth, including in 72% of infundibulae with a normal occlusal surface, confirming the poor relationship between the presence of occlusal caries and deeper infundibular cemental lesions, especially in younger horses.

Infundibular lesions observed on standard CT included two main patterns of developmental cemental hypoplasia, i.e., apical cemental hypoplasia involving the full width of the infundibulum more apically and central linear hypoplasia involving the more central part of most of the infundibulum through most of its length, or combinations of these two types of cemental hypoplasia. Some “normal-sized” (circa 1 mm diameter) central vascular channels expanded subocclusally then became central linear hypoplastic defects, casting doubt what may be considered as “normal” dimensions, if any, of these central cemental defects. Acquired infundibular caries can later affect developmental cemental defects when due to occlusal wear, they become exposed on the occlusal surface.

## Ethics Statement

This study was approved by the Ethical Review Committee of the Royal (Dick) School of Veterinary Studies and the Roslin Institute, The University of Edinburgh on 12^th^ February 2012.

## Author Contributions

AH contributed to the study design and execution, data analysis and interpretation, and manuscript preparation. SS contributed to study design and execution and interpretation and manuscript preparation. PD contributed to the study design and execution, data interpretation, and manuscript preparation.

### Conflict of Interest Statement

The authors declare that the research was conducted in the absence of any commercial or financial relationships that could be construed as a potential conflict of interest.
